# Chronic pain and sex-differences; women accept and move, while men feel blue

**DOI:** 10.1371/journal.pone.0175737

**Published:** 2017-04-25

**Authors:** Graciela S. Rovner, Katharina S. Sunnerhagen, Ann Björkdahl, Björn Gerdle, Björn Börsbo, Fredrik Johansson, David Gillanders

**Affiliations:** 1 Rehabilitation Medicine, Institute of Neuroscience and Physiology, University of Gothenburg at Sahlgrenska Academy, Gothenburg, Sweden; 2 Division of Rehabilitation Medicine, Department of Clinical Sciences, Karolinska Institutet Danderyd University Hospital, Stockholm, Sweden; 3 Department of Neurobiology, Care Sciences and Society, Division of Physiotherapy, Karolinska Institutet, Stockholm, Sweden; 4 Ersta Skondal University College, Campus Bracke, Gothenburg, Sweden; 5 Pain and Rehabilitation Centre, and Department of Medical and Health Sciences, Linkoping University, Linkoping, SE-581 85 Linköping, Sweden; 6 Division of Community Medicine, Department of Medical and Health Sciences, Linkoping University, Linkoping, Sweden; 7 Clinical Department of Rehabilitation Medicine, County Hospital Ryhov, Jonkoping, Sweden; 8 Medical library, Danderyd University Hospital, Stockholm, Sweden; 9 School of Health in Social Science, University of Edinburgh, Edinburgh, United Kingdom; Southeast University Zhongda Hospital, CHINA

## Abstract

**Purpose:**

The aim of this study is to explore differences between male and female patients entering a rehabilitation program at a pain clinic in order to gain a greater understanding of different approaches to be used in rehabilitation.

**Method:**

1371 patients referred to a specialty pain rehabilitation clinic, completed sociodemographic and pain related questionnaires. They rated their pain acceptance (CPAQ-8), their kinesiophobia (TSK), the impact of pain in their life (MPI), anxiety and depression levels (HAD) and quality of life scales: the SF-36, LiSat-11, and the EQ-5D. Because of the large sample size of the study, the significance level was set at the *p* ≤.01.

**Results:**

Analysis by *t-*test showed that when both sexes experience the same pain severity, women report significantly higher activity level, pain acceptance and social support while men report higher kinesiophobia, mood disturbances and lower activity level.

**Conclusion:**

Pain acceptance (CPAQ-8) and kinesiophobia (TSK) showed the clearest differences between men and women. Pain acceptance and kinesiophobia are behaviorally defined and have the potential to be changed.

## Introduction

It is well known that the prevalence of chronic musculoskeletal pain is higher among women, both in community and treatment seeking cohorts [1–5]. In addition, response to treatment differs between sexes [6–10]. In this article, the term ‘sex’ is chosen only because in this study the data inform about the patients’ biological sex, not about their gender [11–13]. Basic research in laboratory environments show that women are more sensitive to pain [14–16], report higher pain intensity and more often report widespread pain [17, 18, 19]. However, despite a large volume of laboratory-based research in this area, a consistent pattern of sex differences in pain sensitivity, expression and impact has not yet emerged [20]. What it is known from clinical studies is that women use more analgesic medication [21] and are more sensitive to both dosage and type of medication [22–24], but other than that, basic research has not been successfully linked to meaningful differences that can influence the design of pain rehabilitation packages [25].

An inconsistency is also found regarding what extent there are sex differences with respect to different psychological factors [20, 26]. For example, some studies report higher anxiety levels among men [27], while others report higher anxiety among women [9, 28]. Depression is twice as common in women as in men [29]. Men, on the other hand, report lower physical and psychological quality of life [30] and tend to score higher than women on specific aspects of anxiety, such as kinesiophobia (movement avoidance) [26].

Clinical and rehabilitation relevant measures are those sensitive to sexes’ potentially differential rehabilitation needs. These differences may also support decision making, to assess and allocate patients into different rehabilitation packages, potentially designed for the different sexes’ needs [31].

The aim of this study was to explore differences in baseline outcome variables between male and female patients referred to a cognitive behaviorally based rehabilitation program at a multidisciplinary pain center.

## Materials and methods

### Participants

The participants in this study were 1371 patients with chronic musculoskeletal non-malignant pain consecutively referred to the Pain and Rehabilitation Centre, University Hospital, Linköping, Sweden between 2009 and April 2011. The clinic specializes in multidisciplinary pain rehabilitation and pain assessments and as such taking part in the Swedish National Quality Registry of Pain Rehabilitation (SQRP).

### The Swedish National Quality Registry of Pain Rehabilitation (SQRP)

To ensure quality of pain care services and in order to develop evidence-based methods in pain rehabilitation, the SQRP has been implemented in around 40 specialty clinics in Sweden. The registry is used both for quality control in health-care management and for clinical research. The data collected in SQRP can be divided into two sections: 1) Sociodemographic data and 2) psychometric instruments.

The instruments included in the SQRP are completed before the first assessment (pre-rehab), after rehabilitation (post-rehab) and at a 1-year follow up. This study used the pre-rehab data to explore sex differences.

### Ethics

The questionnaires are normally sent by mail to the participants who then send them back by post before the first visit, together with their informed written consent. Ethical approval was obtained from the Regional Ethics Board in Gothenburg (815–12).

### Sociodemographic data

The SQRP includes the following socio-demographic data: education, work status, sick leave or insurance situation.

### Psychometric instruments

#### Pain characteristics

The following pain related data were gathered a) pain severity (from the MPI, read below under MPI), b) the duration of pain and duration of persistent pain in years; c) quantity of regions with pain (between 0 and 36) and d) location of worst pain, or the alternative to mark widespread pain.

#### Hospital Anxiety and Depression scale

(HAD; [32]). The HADS rates the severity of depression and anxiety symptoms separately in two self-administrated subscales, which include 7 items. The scale is designed for non-psychiatric hospital settings and excludes items that might reflect somatic complaints. Each item has four Likert’s responses ranging from 0 (no complaints) to 3, yielding a maximum score of 21 for each component. A score of <7 in a component is taken as a normal result; a score of 8–10 indicates mild symptoms and complaints; >10 indicates moderate, and >14 severe symptoms [33–35]. The Swedish translation has shown acceptable psychometric properties [36]. In the current study, the internal consistency of the anxiety sub-scale was .77 and.76 for the depression subscale.

#### The short form 36 survey questionnaire for chronic pain

(SF-36; [37]) The SF-36 measures Health Related Quality of Life by assessing the ability of the patient to function, the impact of emotions on daily functioning, and the impact of pain on daily functioning. It includes 36 questions that yield an 8-scale profile of functional health and well-being scores: Physical Functioning (PF), Role Physical (RP), Bodily Pain (BP), General Health (GH), Vitality (V), Social Functioning (SF), Role Emotional (RE) and Mental Health (MH) as well as two psychometrically-derived summary clusters, the Physical Component and the Mental Component Summary (PCS and MCS) [38]. The internal consistency of the eight scales of the SF-36 in the Swedish norm is between .79 to .93, comparable to US and UK scales [39] and in this study the Alpha were between .70 to.87.

#### EuroQuol, quality of life measure

(EQ-5D; [40, 41–43]) is a generic measure of health-related quality of life, covering five dimensions: mobility, self-care, usual activities, pain/discomfort, and anxiety/depression. In addition, there is a global self-rating of QoL according to a 100-point scale, a thermometer-like visual analogue scale with defined end points (high values indicate good health and low values indicate poor health). The EQ-5D has been well-validated across a number of countries, settings and conditions. It has good psychometric properties and the Cronbach alpha for this study was .58. The EuroQol allows the calculation of a total index score, with higher values indicating a better health-related QoL.

#### Life satisfaction questionnaire (LiSat-11)

[44, 45] consists of 11 items relating to satisfaction with life, activities and participation (Likert scale 1–6) relating to health status, the vocational and financial situation as well as relationships. High scores reflect greater level of life satisfaction [46].

#### Multidimensional Pain Inventory (MPI)

The MPI has been validated and translated into several languages. The Swedish version (MPI-S), includes 61 items, has good internal consistency, with a Cronbach’s alpha of between 0.6 and 0.9 [47–50]. Alpha in the current study was .79. The original MPI has three sections and 12 scales. Part 1 contain five scales: Pain severity; Pain Interference; Perceived Life Control; Affective Distress; and Social Support. Part 2 assesses the perception of responses from significant others to displays of pain and suffering and consists of three scales: Punishing Responses; Solicitous Responses; and Distracting Responses. Part 3 measures the extent to which patients engage in various activities and these four scales are combined in a composite scale labeled general activity index (MPI-GAI). In the Swedish validation, some of the scales has been excluded [51, 52], but in the SQRP these are still included, thus also reported in this study.

#### The Chronic Pain Acceptance Questionnaire 8-items *(CPAQ-8)*

[53] measures acceptance behaviours and attitudes towards pain. This short form was extracted from the long form (20-items) (CPAQ; [54, 55]). The CPAQ-8 has demonstrated good reliability and validity both in English and Swedish[56–58]. Pain acceptance is operationalized with two main classes of behavior (subscales): 1) ‘Activity Engagement’ (‘AE’, score range: 0–24), which involves continuing to engage in personally valued activity despite the presence of pain and 2) ‘Pain Willingness’ (‘PW’, score range: 0–24), the capacity to be open to pain without struggling against it, or unproductive efforts to control it. This willingness and openness to discomfort is functional when it is in the service of living a personally valued life. The eight items are rated on a scale from 0 (never true) to 6 (always true). In the current study, the internal consistency of the AE sub-scale was .88 and .74 for the PW.

#### The Tampa Scale for Kinesiophobia (TSK)

[59] measures pain-related fear of movement [60]. The items are rated on a 4-point Likert scale ranging from ‘‘strongly disagree” to ‘‘strongly agree.” The total score has a range from 17–68 where scores higher than 36 for women and 38 for men indicate high pain-related fear [61]. Male patients score somewhat higher than female patients [26]. The TSK has proven to be a reliable assessment tool for chronic pain [60, 62, 63]; the factor structure has been demonstrated to be stable across pain diagnoses and nationalities [64].

### Statistics

All analyses and procedures were carried out in the statistical software package IBM SPSS 19.0.0.1 for Mac. Background data is presented descriptively with means, SDs, and comparisons between the sexes (Tables 1 and 2). Descriptive statistics (means and *SD*s) of the outcome variables, the psychometric instruments, were computed for the sample and were grouped according to sex. Each instrument was first reported using descriptive statistics, and then comparisons were carried out between the sexes for each instrument using Chi Squared and t-tests (two-tailed).

**Table 1:** Percentages and differences between men and women in respect to their sociodemographic data and presence of widespread pain.

Variable (*N*)	All *(%)*	Women *(%)*	Men *(%)*	Sig. *p*
Born in Sweden (*1368*)	79.6	83.0	72.2	< .001*
University education (*1307*)	17.1	18.0	15.3	.219
Widespread pain^1^ (*1283*)	31.0	35.1	21.8	< .001*
Sickness benefit 100% (*1368*)	13.9	13.8	14.1	.866
Time-limited sickness benefit 100% (*1368*)	5.6	6.0	4.6	.310
Working/studying 100% (*1331*)	25.3	23.6	29.0	.035
Working/studying to some extent (*1331*)	37.3	39.4	32.9	.030

*p ≤ .001

^1^ the patients reported that their pain was not localized in one area but spread over several body regions.

**Table 2:** Differences between men and women regarding their age, their pain severity, duration, persistency and location as well as regarding their sick-leave-time.

Variable *(N)*	All		Women		Men		Sig.	Effect
	M	SD	M	SD	M	SD	*p*	*Size*^*1*^
Age *(1368)*	47	15	45	15	51	14	< .001*	-0.41
Pain Severity MPI (range 0–6) *(1217)*	4.50	1.04	4.54	0.99	4.43	1.14	.123	0.11
Pain duration (days) *(1145)*	2953	3319	2939	3046	2982	3831	.850	-0.01
Persistent pain duration (days) *(936)*	2425	3048	2304	2559	2677	3866	.127	-0.12
N:o of pain locations (0–36) *(1317)*	13	8	14	8	10	8	< .001*	0.50
Days since last job *(574)*	2337	3200	2396	3480	2216	2542	.530	0.06

*p ≤ .001

^1^ Effect Sizes Cohen’s *d*. A negative value indicates that women’s values are lower.

Because of the large sample size of the study, stringent *p*-values were chosen, to reduce the likelihood of Type 1 error. In this study, results that were significant at the *p* ≤.01 were considered significant, and at the *p* ≤. 001 were considered "highly" significant. Significances between .05 and .01 were seen as ‘tendencies’ that if relevant could be discussed. Cohen’s Effect Sizes were calculated and a *d*>.8 was considered as large (8/10 of a standard deviation unit), a *d* between .5 and .7, as moderate and between .2 and .4 as small [65]. Cases with missing data were excluded on a pairwise basis and the *n* of each analysis is stated in the results.

## Results

Background descriptive is presented in Tables 1 and 2 and show some significant differences between sexes. Of the 1371 patients, 68.4% were women and 35% of them suffered from widespread pain while the majority of men (78%) suffered from localized pain *χ*^*2*^ (one, *n* = 1283) = 22.63, *p* = .000 (**Table 1)**. The patients have had pain for an average of 8 years, and they suffered of continuous pain for an average of 6 years. However, the variation among the patients was larger than the means (*SD* = 9 and 8.3 respectively) showing the heterogeneity of the total group. As seen in **Table 2,** women reported a greater number of pain locations than men *t* (1316) = 8.63, *p* = < .0001, *d* = .50. The magnitude of the differences in these means was large (mean difference = 4.032, 99% *CI*: 2.82 to 5.24).

Women were significantly younger than men (Age *M* = 45 vs. men’s *M* = 51) *t* (1367) = -6.52, *p* < .001, *d* = .41 (**Table 2)** and more were born in Sweden (Table 1).

A quarter of participants were working full time and one third were receiving full sickness benefit. Most of the men that worked, were working full time *χ*^*2*^ (1, *n* = 1331) = 7.012, *p* = .030, while most women were working to some extent *χ*^*2*^ (1, *n* = 1331) = 6.684, *p* = .035 (**Table 1**) and 41% (*n* = 574) had been unemployed for, on average, 6 years (**Table 2**).

Mental Health was measured with HAD, EQ-5D and SF-36. The majority of men (42%) and women (46%) scored low levels of anxiety (HAD scored under 8). Almost the same amount of men (38%) and women (34%) scored between 11 and 21, which is considered indicative of a case of anxiety. The remaining 20% of both sexes scored borderline levels (scores of 8 to 10), showing no statistically significant difference in anxiety according to HAD. The same is true for depression, when almost 48% of women and 41% of men scored low levels, around 24% of both sexes scored borderline levels. More men than women (34% vs 30%) scored at a level of depression. No differences were in the single item from EQ-5D item about Anxiety and depression. However, according to the Mental Health subscale of SF-36, men scored significantly lower than women, indicating that men were experiencing more problem with their mental health than women *t*(1109) = 2.78, *p* = .007, *d =* .18 (mean difference = 4.08, 99% *CI*: 0.18 to 7.98) (**Table 3**).

**Table 3:** Mean and SD for the whole population and separately for women and men for mood (HAD), quality of life (EQ-5D, SF-36 and LiSat-11), pain impact (MPI), pain acceptance (CPAQ) and in kinesiophobia (TSK). The effects sizes and significance (*p*) are shown for differences between men and women.

	Sub-scale (min-max) (*N*)	All		Women		Men		Sig.	Effect
		M	SD	M	SD	M	SD	*p*	Sizes^1^
**HAD**	Anxiety (0–21) (1203)	8.73	5.06	8.62	5.01	8.99	5.16	.245	-0.07
	Depression (0–21) (1208)	8.42	4.66	8.25	4.63	8.80	4.71	.056	-0.12
**MPI**	Pain Severity (0–6) (1217)	4.50	1.04	4.54	0.99	4.43	1.14	.123	0.11
	Pain interference (0–6) (1216)	4.36	1.15	4.41	1.14	4.25	1.35	.043	0.13
	Life control (0–6) (1216)	2.62	1.24	2.64	1.21	2.56	1.31	.296	0.06
	Affective distress (0–6) (1216)	3.40	1.44	3.43	1.41	335	1.49	.359	0.06
	Social support (0–6) (1216)	4.18	1.46	4.20	1.40	4.15	1.60	.594	0.03
	Punishing responses (0–6) (1213)	1.61	1.41	1.61	1.43	1.63	1.37	.806	-0.01
	Solicitous responses (0–6) (1213)	2.59	1.68	2.64	1.64	2.50	1.77	.192	0.08
	Distracting responses (0–6) (1213)	2.17	1.42	2.19	1.39	2.14	1.49	.544	0.04
	General Activity Level (0–6) (1213)	2.29	0.94	2.36	0.92	2.16	1.00	< .001*	0.21
**EQ**-**5D**	1. Mobility (1–3) *(1205)*	1.66	0.50	1.66	0.50	1.66	0.50	.966	0.00
	2. Self-care (1–3) *(1211)*	1.24	0.46	1.23	0.45	1.26	0.49	.199	-0.06
	3. Usual activities (1–3) *(1209)*	1.97	0.68	1.97	0.65	1.97	0.72	.966	0.00
	4. Pain/discomfort (1–3) *(1213)*	2.64	0.50	2.62	0.50	2.67	0.50	.105	-0.10
	5. Anxiety/depression (1–3) *(1222)*	1.95	0.63	1.94	0.63	1.96	0.63	.565	-0.03
	EQ-VAS- Health (0–100) *(1219)*	39.84	20.94	40.11	20.78	39.30	21.29	.539	0.04
	EQ-5D Index (max 1) *(1083)*	0.25	0.32	0.26	0.32	0.22	0.32	.143	0.13
**SF**-**36**	Physical functioning (0–100) *(1115)*	48.74	24.42	48.38	23.78	49.47	25.68	.010*	-0.04
	Role-Physical (0–100) *(1080)*	15.65	29.03	15.05	28.49	16.90	30.11	.162	-0.06
	Bodily Pain (0–100) *(1114)*	23.16	15.24	23.25	15.13	22.98	15.47	.876	0.02
	General Health (0–100) *(1096)*	39.37	21.03	39.74	21.36	38.62	20.35	.165	0.05
	Vitality (0–100) *(1111)*	27.03	20.64	26.52	20.46	28.08	20.99	.785	-0.07
	Social Functioning (0–100) *(1120)*	47.76	26.96	47.36	26.53	48.57	27.82	.293	-0.05
	Role-Emotional (0–100) *(1067)*	40.29	43.63	40.19	43.72	40.49	43.51	.885	-0.01
	Mental Health (0–100) *(1110)*	55.19	23.08	56.53	22.36	52.47	24.27	.007*	0.18
	Physical Component (0–80) *(1031)*	28.02	8.71	27.73	8.54	28.63	9.03	.297	-0.10
	Mental Health Comp. (0–80) *(1031)*	35.62	12.91	35.88	12.89	35.08	12.96	.914	0.06
**LiSat-11-** Satis-faction with…	Life as a whole (1–6) *(1239)*	3.46	1.90	3.60	1.35	3.31	1.40	< .001*	0.21
	Vocational situation (1–6) *(1175)*	2.88	2.47	2.87	1.61	2.88	1.65	.896	-0.01
	Financial situations (1–6) *(1241)*	3.39	2.06	3.30	1.55	3.48	1.64	.057	-0.11
	Spare time (1–6) *(1233)*	2.95	2.06	3.01	1.40	2.89	1.46	.155	0.08
	Friends/acquaintances (1–6) *(1246)*	3.66	1.89	3.77	1.45	3.54	1.45	.010*	0.16
	Sexual life (1–6) *(1213)*	2.89	2.47	3.05	1.62	2.73	1.58	< .001*	0.20
	Personal ADL (1–6) *(1248)*	4.09	1.86	4.15	1.51	4.03	1.52	.202	0.08
	Family life (1–6) *(1191)*	4.06	2.19	4.35	1.58	3.76	1.97	< .001*	0.35
	Partner relationship (1–6) *(1135)*	3.70	2.76	3.86	2.17	3.54	2.31	.027	0.14
	Somatic health (1–6) *(1246)*	2.31	2.02	2.31	1.29	2.30	1.26	.896	0.01
	Psychological health (1–6) *(1247)*	3.52	1.93	3.56	1.46	3.47	1.54	.349	0.06
**CPAQ-8**	Activities Engagement (0–24) *(1325)*	12.83	9.49	13.46	9.76	11.45	8.73	< .001*	0.21
	Pain Willingness (0–24) *(1086)*	9.51	5.35	10.08	5.27	8.36	5.34	< .001*	0.33
**TSK**	Kinesiophobia (17–68) *(869)*	41.51	9.30	39.50	8.83	43.52	9.63	< .001*	-0.44

**Abbreviations: HAD:** The Hospital Anxiety and Depression Scale, **MPI:** The Multidimensional Pain Inventory, **EQ-5D:** EuroQuol, quality of life measure **SF36**, Medical Outcome Study Short Form 36; **CPAQ-8:** Chronic Pain Acceptance Questionnaire- 8 items; **LiSat-11:** Life Satisfaction questionnaire; **TSK:** Tampa Scale for Kinesiophobia

*p≤ .001

^1^ Effect Sizes Cohen’s *d*. A negative value indicates that women’s values are lower.

According to CPAQ, women scored significantly higher levels of Activity Engagement than men *t* (1323) = 3.59, *p* = < .001, *d =* .21 (mean difference = 2.01, 99% *CI*: .56 to 3.45), and Pain Willingness *t* (1084) = -5.03, *p* = < .001, *d =* .33 (mean difference = 1.72, 99% *CI*: .84 to 2.61) (**Table 3**).

Women scored less on physical function (SF-36 PF) than men, *t* (1114) = 12.04, *p* = < .001, *d =* .04 (mean difference = 0.20, 99% *CI*: 0.084 to 1.30) while men reported significantly higher levels of kinesiophobia (TSK) than women *t*(867) = -6.15, *p* = < .001, *d =* .44 (mean difference = -3.99, 99% *CI*: -5.71 to -2.26). That women scored higher in general activity level as reflected by the MPI, *t* (1212) = 3.31, *p* < .001, *d =* .21 (mean difference = 0.20, 99% *CI*: 0.04 to 0.35). The effect sizes of these differences are however low.

According to LiSat-11, women stated higher satisfaction with: life as a whole friends and acquaintances as well as greater satisfaction with their family life, and their sexual life (See LiSat-11 **Table 3**).

## Discussion

The main results were that at the same pain severity, women report significantly higher activity level, pain acceptance and social support while men report higher kinesiophobia, mood disturbances and lower activity level.

In the present population, there were fewer men than women born in Sweden were as well as men being older. However, the total group of patients included in this study was representative when compared with a European epidemiological study [1] in terms of age and sex (middle-aged women), and pain duration (> 7 years) and with the pain population attending specialty pain care clinics in Sweden [66]. The overall poor quality of life of the patients in this study, according to the SF-36, was consistent with previously published pain studies [67–69] but distinctively lower than the Swedish population norm [38].

Some of the differences already known between men and women with chronic pain were confirmed by this study. More women than men seek rehabilitation, they present with pain in more body areas, and or widespread pain, while men report having more often localized pain. Among the group of patients in this study, more men were born outside Sweden and they were older than women (**Tables 1 and 2)**. These demographic differences may or may not be clinical significant, while differences in how they react and behave while in pain, might have a clinical impact in rehabilitation.

Women in this study reported higher pain acceptance levels and being less afraid of pain. They reported higher activity level (MPI) and activity engagement (CPAQ-8), despite the fact that they have the same level of pain, the same severity of symptoms, the same discomfort and the same somatic health as men. However, men scored having higher physical function (SF-36) but also struggling with psychological issues, such as mood disturbances (SF-36). Even though women had pain in several regions (widespread pain, see **Table 2**) they expressed being more satisfied with their social life (intimate, private, family and friends) and with life in general (Li-Sat11). This capacity for positivity has also been found in a previous study, where it has been linked to greater social support among women with chronic pain, compared to men [70].

Although initially developed for use with chronic lower back pain patients and later validated in other musculoskeletal pain populations, recent studies suggest that it is a valid measure of pain-related fear of movement in Swedish [63] and for heterogeneous chronic pain samples. It was interesting to find that women scored significantly lower in kinesiophobia than men. Women also scored having a higher level of activity even though they reported pain in multiple body areas (**Table 2),** which was consistent with prior studies [14, 18, 22]. Also consistent with prior investigation, men‘s statistically significant higher levels of kinesiophobia is also consistent with prior studies [60, 71, 72]. In this study both men and women score above the kinesiophobia cut off. But why men are significantly more avoidant to move than women can depend upon social norms, higher expectations or a deeper concern about losing work capacity or productivity as a result of re-injury, thus with a risk of having a sedentary life. It has been demonstrated that kinesiophobia has shown to be a strong predictor for pain disability and pain chronicity in the longer term [73] associated with higher rates of sick-leaves [74], pain vigilance and disability as well as decreased physical activity levels [75–77].

One limitation to the current study is that the large sample size can lead to results that are overall small in magnitude, but nevertheless, highly statistically significant. These results must be treated with suitable caution. To counteract this, we made the value of *p* to be accepted as significant to be lower than convention, and we provided effect sizes as a guide to the overall magnitude of effect, uninfluenced by sample size. The effect size was in some cases low, which is an indication of that the significance found may not be a meaningful difference. Another limitation are the potential flaws that self-reports can contain. This study may have benefited from gathering observational measures, such as physical capacity, activity level, strength, sedentary-time, etc. in order to see how the self-reported activity level correlates with the factual one.

It is important to interpret the significances in a frame of studying a very heterogeneous group indicated by large SD in some areas such duration of pain and number of pain location. This heterogeneity may be the cause of the relatively low effect sizes despite some significant differences. If the females and males were further stratified or clustered by rehabilitation relevant indicators potentially other and more clinical relevant differences might be identified. A limitation is that this has not be done, since it would have required a larger sample. Another limitation in this this study is that due to its exploratory and cross-sectional design, no causal inferences can be made, nor can it be established that the found differences necessarily influence treatment response. However, if outcomes were potentially sex-dependent, treatment may be more effective by differentially targeting the processes supporting the different needs, expectations and coping mechanisms of each of the sexes. It could be a possibility in the future to use the results from questionnaires to allocate patients into different rehabilitation packages, depending on the needs. Is looks as if the needs of the males and female in general are different. Further research is needed in order to investigate to what extent the differences we found are due to disparities in assessment modalities, practitioners’ attitude or referral patterns, knowledge and/or stigmatization that the patient has met before [78] or after referral [4].

To conclude, this study indicate differences between men and women regarding symptomatology and their reaction or handling these. The results give some suggestions that the sexes differ in how they accept their pain, which could potentially be useful to consider while selecting patients for rehabilitation programs (or designing programs adapted to the sexes).
